# Supplementary material for: The Effect of Vinyasa Yoga Practice on the Well-Being of Breast-Cancer Patients during COVID-19 Pandemic
Source: Int J Environ Res Public Health. 2023 Feb 20;20(4):3770. doi: 10.3390/ijerph20043770 (PMC9967391; doi:10.3390/ijerph20043770)
Supplement: Supplementary file 1 [file ijerph-20-03770-s001.zip › blank survey form.pdf]

Survey

Before practice

Do you practice yoga?
Yes, regularly
Yes, sometimes
I practiced in the past
I never practiced yoga

What are your motivations to practice yoga?
better stress management
improved quality of sleep
specific health needs
the desire to stretch the body
build strength
meditation/spiritual needs
the need to integrate psyche and body
the need to increase vitality

How do you assess your overall health during the past week?
Very bad 1 2 3 4 5 6 7 excellent

Does a long walk make you tired?
Never 1 2 3 4 Very often

Does a short walk outside the house make it difficult for you?
Never 1 2 3 4 Very often

Do you need help eating, dressing, washing yourself or using the toilet?
Never 1 2 3 4 Very often

Have you been restricted from doing your job or other daily activities in the past week?
Never 1 2 3 4 Very often

Have you been limited in pursuing your hobbies or other pleasures in the past week?
Never 1 2 3 4 Very often

Have you had any pains in the past week?
Never 1 2 3 4 Very often

Have you needed any rest in the past week?
Never 1 2 3 4 Very often

Have you experienced difficulty sleeping in the past week?
Never 1 2 3 4 Very often

Have you felt any weakness in the past week?
Never 1 2 3 4 Very often

Have you been tired in the past week?
Never 1 2 3 4 Very often

Has pain interfered with your daily activities in the past week?
Never 1 2 3 4 Very often

Have you felt tense in the past week?
Never 1 2 3 4 Very often

Have you been worried in the past week?
Never 1 2 3 4 Very often

Have you felt irritable in the past week?
Never 1 2 3 4 Very often

Have you felt depressed in the past week?
Never 1 2 3 4 Very often

Have you had difficulty remembering in the past week?
Never 1 2 3 4 Very often

In the past week, has your health condition or treatment caused you financial hardship?
Never 1 2 3 4 Very often

Have recent worries kept you up at night?
Never 1 2 3 4 Very often

Have you been able to enjoy your usual daily activities lately?
Never 1 2 3 4 Very often

I would very much like to ask you to choose the answer that best describes your well-being during the last month.

A
I'm not sad or depressed
I often feel sad and depressed
I am experiencing constant sadness, depression and I cannot free myself from these experiences
I am constantly so sad and unhappy that it is unbearable

B
I'm not too worried about the future
I often worry about the future
I'm afraid that nothing good awaits me in the future
I feel that the future is hopeless and nothing will change that

C
I'm satisfied with myself
I'm not satisfied with myself
I feel self-loathing
I hate myself
D
I don't cry more than usual
I cry more often than I used to
I still want to cry
I'd like to cry, but I'm not able to

E

I'm no more nervous than I used to be
I am constantly nervous and irritable
I am constantly nervous and irritable
Everything that used to irritate me has become indifferent

F
I sleep well, as usual
I sleep worse than I used to
In the morning, I wake up 1-2 hours too early and find it difficult to get back to sleep again
I wake up a few hours too early and can't get to sleep

Age
Date of birth

Sex
…….

After practice

Did you notice the effects of yoga practice?
Improve health
Improve body flexibility
Muscle gain
Improving mental health
Stress relief
Reduction of sleep problems
Alleviation of digestive/stomach discomforts
Improved respiratory capacity
Fatigue reduction

How do you assess your overall health during the past week?
Very bad 1 2 3 4 5 6 7 excellent

Does a long walk make you tired?
Never 1 2 3 4 Very often

Does a short walk outside the house make it difficult for you?
Never 1 2 3 4 Very often

Do you need help eating, dressing, washing yourself or using the toilet?
Never 1 2 3 4 Very often

Have you been restricted from doing your job or other daily activities in the past week?
Never 1 2 3 4 Very often

Have you been limited in pursuing your hobbies or other pleasures in the past week?
Never 1 2 3 4 Very often

Have you had any pains in the past week?
Never 1 2 3 4 Very often

Have you needed any rest in the past week?
Never 1 2 3 4 Very often

Have you experienced difficulty sleeping in the past week?
Never 1 2 3 4 Very often

Have you felt any weakness in the past week?
Never 1 2 3 4 Very often

Have you been tired in the past week?
Never 1 2 3 4 Very often

Has pain interfered with your daily activities in the past week?
Never 1 2 3 4 Very often

Have you felt tense in the past week?
Never 1 2 3 4 Very often

Have you been worried in the past week?
Never 1 2 3 4 Very often

Have you felt irritable in the past week?
Never 1 2 3 4 Very often

Have you felt depressed in the past week?
Never 1 2 3 4 Very often

Have you had difficulty remembering in the past week?
Never 1 2 3 4 Very often

In the past week, has your health condition or treatment caused you financial hardship?
Never 1 2 3 4 Very often

Have recent worries kept you up at night?
Never 1 2 3 4 Very often

Have you been able to enjoy your usual daily activities lately?
Never 1 2 3 4 Very often

I would very much like to ask you to choose the answer that best describes your well-being during the last month.

A
I'm not sad or depressed
I often feel sad and depressed
I am experiencing constant sadness, depression and I cannot free myself from these experiences
I am constantly so sad and unhappy that it is unbearable

B
I'm not too worried about the future
I often worry about the future
I'm afraid that nothing good awaits me in the future

I feel that the future is hopeless and nothing will change that

C
I'm satisfied with myself
I'm not satisfied with myself
I feel self-loathing
I hate myself
D
I don't cry more than usual
I cry more often than I used to
I still want to cry
I'd like to cry, but I'm not able to

E
I'm no more nervous than I used to be
I am constantly nervous and irritable
I am constantly nervous and irritable
Everything that used to irritate me has become indifferent

F
I sleep well, as usual
I sleep worse than I used to
In the morning, I wake up 1-2 hours too early and find it difficult to get back to sleep again
I wake up a few hours too early and can't get to sleep

Age
Date of birth

Sex
…….
