# Supplementary material for: The Effect of Vinyasa Yoga Practice on the Well-Being of Breast-Cancer Patients during COVID-19 Pandemic
Source: Int J Environ Res Public Health. 2023 Feb 20;20(4):3770. doi: 10.3390/ijerph20043770 (PMC9967391; doi:10.3390/ijerph20043770)
Supplement: Supplementary file 1 [file ijerph-20-03770-s001.zip › Supplementary Figure S1.pdf]

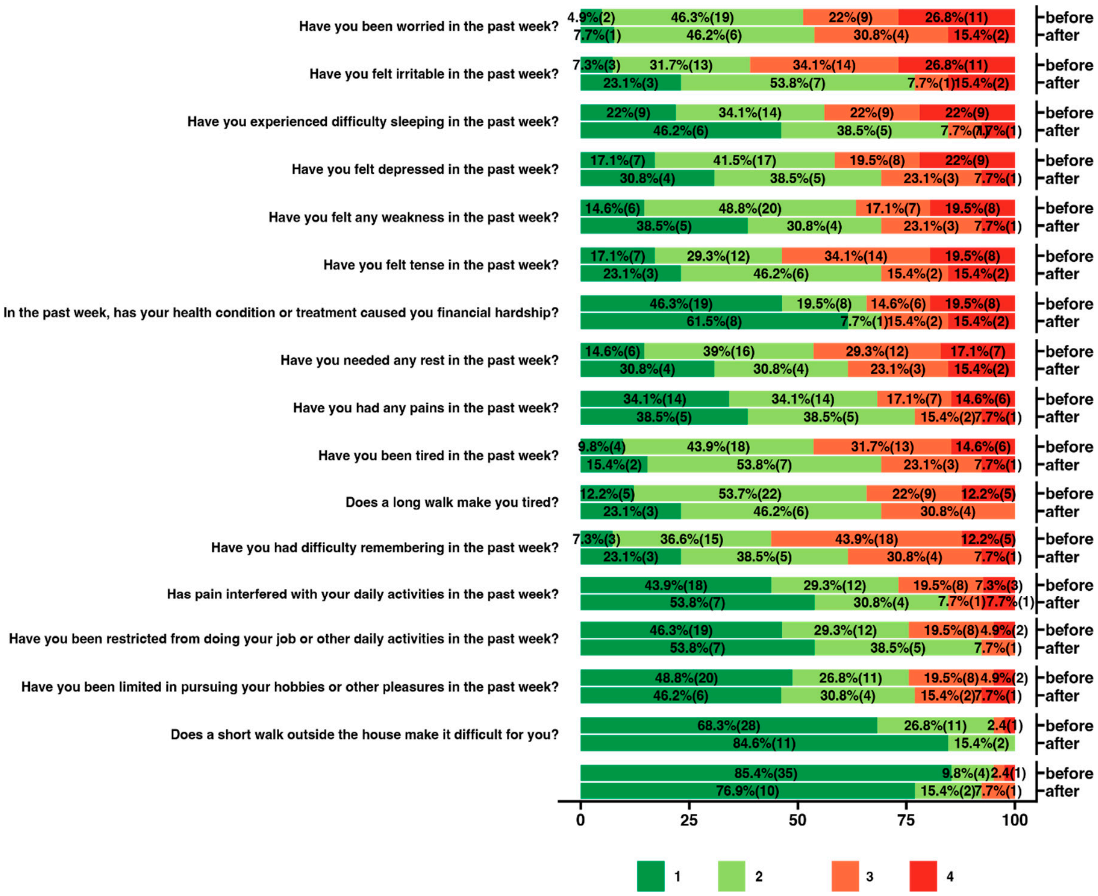

Figure S1. Health problems and ailments of the cancer patients who participated in yoga classes. The number of declared responses (n- shown in brackets) and the percentage distribution (%) were determined based on the results of a survey conducted before and after the yoga course. The survey questions were answered with reference to a numerical scale where 1 means never, 2-sometimes, 3-often and 4 - very often. The questions were ordered according to the answers in which most respondents marked 4 - as very often.
